# Update on the Pharmacological Treatment of Alzheimer’s Disease

**DOI:** 10.2174/157015910790909520

**Published:** 2010-03

**Authors:** Fadi Massoud, Serge Gauthier

**Affiliations:** 1 Department of Medicine, University of Montreal, Centre Hospitalier de l’Université de Montréal (CHUM), Hôpital Notre-Dame, Service de Gériatrie, 1560 Sherbrooke Est, Montreal, Quebec, H2L 4M1; 2 Departments of Neurology & Neurosurgery, Psychiatry, Medicine, McGill University, Alzheimer Disease and Related Disorders Unit, McGill Center for Studies in Aging, Douglas Mental Health University Institute, 6825, boul. LaSalle, Verdun, Montréal (Québec), H4H 1R3

**Keywords:** Dementia, Alzheimer’s, therapy, pharmacological, cholinesterase inhibitor, memantine.

## Abstract

Alzheimer’s disease (AD) is the most common neurodegenerative disorder. Worldwide prevalence of the disease is estimated at more than 24 million cases. With aging of populations, this number will likely increase to more than 80 million cases by the year 2040. The annual incidence worldwide is estimated at 4.6 million cases which is the equivalent of one new case every seven seconds! The pathophysiology of AD is complex and largely misunderstood. It is thought to start with the accumulation of beta-amyloid (Aβ) that leads to deposition of insoluble neuritic or senile plaques. Secondary events in this “amyloid cascade” include hyperphosphorylation of the protein *tau* into neurofibrillary tangles, inflammation, oxidation, and excitotoxicity that eventually cause activation of apoptotis, cell death and neurotransmitter deficits. This review will briefly summarize recent advances in the pathophysiology of AD and focus on the pharmacological treatment of the cognitive and functional symptoms of AD. It will discuss the roles of vascular prevention, cholinesterase inhibitors and an NMDA-antagonist in the management of AD. It will address the issues thought to be related to the lack of persistence or discontinuation of therapy with cholinesterase inhibitors shown in recent studies and some of the solutions proposed. These include setting realistic expectations in light of a neurodegenerative condition and available symptomatic treatments, slowly titrating medications, and using alternate routes of administration. Finally, it will introduce future therapeutic options currently under study.

## INTRODUCTION

The year 2006 marked the centennial of the description by Alois Alzheimer of the first case of the disease that will eventually hold his name. Alzheimer’s disease (AD) is the most common neurodegenerative disorder. Worldwide prevalence of dementia is estimated at more than 24 million cases [44]. The annual incidence worldwide is estimated at 4.6 million cases which is the equivalent of one new case every seven seconds ! [44]. With aging of populations, this number will double every twenty years, likely increasing to more than 80 million cases by the year 2040 [44]. Aging is amongst the major risk factors for the disease. For example, in the Canadian Study on Health and Aging, close to 10% of all individuals aged 65 and older were affected by dementia and this rose to 35% in individuals aged 85 and older [21]. Most cases of dementia are due to AD with or without a cerebrovascular contribution [23]. The global burden of disability associated with dementia in the elderly is believed to be higher than in stroke, musculoskeletal disease, heart disease and cancer. Finally, the annual cost of dementia in the UK (17 billion pounds) is estimated to be higher than the costs of heart disease, stroke and cancer combined [19]. This review will briefly summarize recent advances in understanding the pathophysiology of AD and focus on the pharmacological treatment of cognitive and functional symptoms of the disease. It will discuss the roles of vascular prevention, cholinesterase inhibitors, and an NMDA-antagonist in the management of AD. It will address the issues thought to be related to the lack of persistence or discontinuation of therapy with cholinesterase inhibitors shown in recent studies and some of the solutions proposed. These include setting realistic expectations, slow titration of the medications, and alternate routes of administration. It will introduce future therapeutic options currently under study.

## PATHOPHYSIOLOGY

**Fig. (1):**
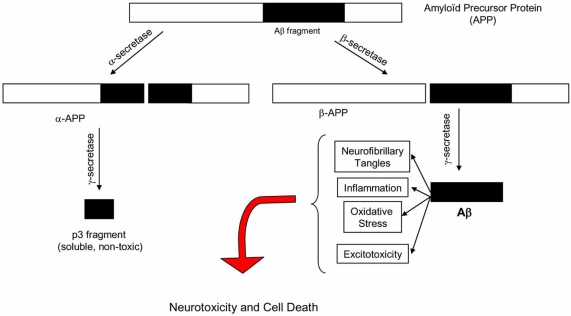
Pathophysiology of Alzheimer ’s Disease.

The pathophysiology of AD is complex and incompletely understood. Several hypotheses have been suggested to explain the series of events that eventually lead to the clinical manifestations of the disease. Presently, the two main competing hypotheses are the amyloid-cascade hypothesis and the hyperphosphorylation of protein *tau* hypothesis, which are ardently supported by the so-called “baptists” and “tauists” respectively [92]. These hypotheses are represented histologically by the two hallmarks that are still used today to reach a definite diagnosis of AD: amyloid plaques and neurofibrillary tangles (NFT) [16, 17]. In AD, amyloid plaques are found in the entorhinal cortex, the hippocampus and neocortical areas [37, 111, 131]. These plaques are also observed in the brains of normal aged individuals and do not correlate with progression of dementia. Neurofibrillary tangles are deposited in a hierarchical and systematic fashion that correlates closely with cognitive decline in AD [7, 18, 67]. They start in the entorhinal cortex and sequentially spread to the hippocampus, the rest of the temporal lobe, the association areas of the prefrontal and parietal cortices, and eventually reach all neocortical areas [35]. Neurofibrillary tangles are also observed in other neurodegenerative disorders. Several arguments support the amyloid-cascade hypothesis as the predominant one. In humans, amyloid precursor protein (APP) found on chromosome 21undergoes cleavage by three enzymatic complexes: α-secretase, β-secretase, and γ-secretase [111, 112] (Fig. **1**). Sequential cleavage of APP by α-secretase and γ-secretase leads to the production of a small, non-toxic, and soluble peptide, referred to as p3. The sequential cleavage of APP by β-secretase and γ-secretase leads to the production of the insoluble beta-amyloid protein that deposits into plaques. Beta-amyloid (Aβ) is neurotoxic *in vitro* [27, 81, 89]. The mouse model of AD over-expressing the Amyloid Precursor Protein (APP) gene shows amyloid plaques similar to the ones found in the brains of AD patients. These mice also display short-term memory and learning deficits on specifically designed tests [66]. In humans, all individuals with clinical AD have abnormal deposits of Aβ (diffuse or neuritic plaques) [59]. Individuals with Down’s syndrome, who have three copies of chromosome 21, almost invariably express clinical and pathological features of AD by mid-life [62]. All mutations described in the rare autosomal-dominant forms of AD affect APP and γ-secretase and are associated with increased deposition of Aβ [3, 75, 83, 107]. Apolipoprotein E4, the main genetic risk factor for sporadic AD, is associated with increased production and deposition of Aβ [25, 108]. Generation of specific antibodies against Aβ in individuals with AD has been associated with mild and inconsistent clinical improvement and clearing of amyloid plaques in some patients [55, 62]. According to the amyloid-cascade hypothesis, abnormal generation (or insufficient clearance of Aβ) leads to several secondary events including hyperphosphorylation of the protein *tau* and generation of neurofibrillary tangles, inflammation, oxidation, and excitotoxicity [60, 84, 86]. These events lead in turn to the activation of the apoptotic cascade, neuronal cell death and neurotransmitter deficits. It is the deficit in acetylcholine, and to a lesser extent in norepinephrin and serotonin, that is thought to be responsible for the clinical manifestations of the disease [51]. 

The main alternate hypothesis to the amyloid cascade lends a central role to hyperphosphorylation of the protein *tau*. This protein normally binds and stabilizes microtubules, the main component of the cellular cytoskeleton. In AD, tau is hyperphosphorylated leading to the formation of paired helical filaments (PHF) and eventually NFTs [56, 74, 125]. These structures disrupt neuronal transport and eventually lead to cell death. The specific timing and chronology of the interaction between deposition of Aβ and hyperphosphorylation of tau is still controversial. Animal studies show that the presence of abnormal tau potentiates Aβ toxicity [2, 102]. This is supported by clinicopathological data suggesting that NFTs mediate the effect of amyloid deposition on clinical disease [5]. 

Recent data suggest that cerebrovascular disease plays an important role in the pathophysiology of AD. There are epidemiological, clinical and pathological similarities between AD and vascular dementia. Also, regional brain hypoperfusion leading to hypometabolism, degenerative changes and cognitive impairment have been described in early stages of AD. These principles are known as the Critically Attained Threshold of Cerebral Hypoperfusion (CATCH) theory [30]. According to this controversial hypothesis, regional hypoperfusion is the primary trigger of the degenerative cascade described earlier [1, 31, 32, 33]. Clinicopathological studies published recently show that most cases of dementia are due to a contribution of degenerative and cerebrovascular lesions (so-called “mixed dementia”) [91, 109]. In fact, some studies suggest that as individuals age, the contribution of amyloid plaques and NFTs to clinical dementia decreases [42, 106], and that concomitant cerebrovascular lesions play a crucial role in modulation of the clinical expression of cognitive impairment [118, 126]. 

## PHARMACOLOGICAL TREATMENT

### Clinical Outcomes in Clinical Trials

Alzheimer’s disease progressively impairs cognitive abilities and behaviour, leading to gradual functional decline. Outcome measures used in clinical trials are selected to mirror clinical hallmarks of the disease. Typically, these trials include a combination of measures evaluating cognition, clinicians’ and caregivers’ impression of change, functional abilities and behaviour. Pharmacological treatment of behavioural and psychological symptoms of dementia (BPSD) have been recently reviewed in this journal [77]. We will focus on cognitive, global, and functional outcomes. ***Cognitive outcome measures*** include the *Alzheimer’s Disease Assessment Scale – cognitive subscale* (*ADAS-Cog* - scored from 0-70, a higher number indicating worse performance) [105], the *Mini-Mental Status Examination* (*MMSE* – scored from 0-30, a higher number indicating better performance) [49], and the *Severe Impairment Battery (SIB –* scored 0-100, a higher number indicating better performance) [97]. ***Global measures*** include the *Clinical Global Impression of Change* (*CGIC* – scored from 1 to 7, 4 indicating no change, 1 and 7 indicating significant improvement and significant deterioration respectively)[110], and the *Clinician Interview-Based Impression of Change* (*CIBIC* – scored from 1 to 7 similarly to the CGIC) [72]. ***Functional measures*** include the *Progressive Deterioration Scale* (PDS – scored from 0-100, a higher number indicating better performance) [34], the *Alzheimer’s Disease Cooperative Study Activities of Daily Living inventory* (ADCS-ADL – scored 0-78, a higher score indicating worse performance) [52], and the *Disability Assessment for Dementia scale* (*DAD – *scored 0-100%, a higher number indicating better performance) [54]. 

### Vascular Prevention

Results of clinical trials in primary prevention of AD are contradictory. Three studies have shown that optimal use of antihypertensive agents in systolic and diastolic hypertension as well as after a cerebrovascular event is associated with a reduction in the incidence of cognitive impairment [50, 123, 127]. The recently completed Hypertension in the Very Elderly Study (HYVET) however showed that treatment of individuals 80 years of age or older is associated with a reduction in cardiovascular and cerebrovascuar end-points, but not in cognitive impairment [4, 98]. In two major primary prevention trials, cholesterol-lowering agents in individuals at risk lead to a reduction in cardiovascular and cerebrovascular events but had no benefit on cognitive outcomes [24, 114]. A secondary prevention trial in individuals with mild cognitive deficits (loosely defined as a MMSE between 20-28) comparing two treatments of systolo-diastolic hypertension did not show any difference between the drugs in terms of cognitive outcomes. Individuals with the best improvements in blood pressure measures benefited the most on working memory measures [119]. A prospective observational study shows that treatment of vascular risk factors in individuals with AD is associated with a slower cognitive decline [36]. There are no published intervention trials in tertiary vascular prevention in AD. These results have led the Canadian Consensus Conference on Diagnosis and Treatment of Dementia (CCCDTD) to recommend treatment of high blood pressure as an option in the prevention of cognitive decline (Grade B, Level I recommendation) [12].

### Cholinesterase Inhibitors

Neurochemical studies of neurons from the basal forebrain of individuals with AD have shown a deficit in choline acetyl-transferase leading to a decrease in the production of acetylcholine and cortical cholinergic dysfunction [128]. These studies suggested that acetylcholine replacing or enhancing therapies might be of benefit in individuals with AD. Acetylcholine precursors and cholinergic receptor agonists were used in clinical trials, but abandoned for lack of effectiveness or intolerable side effects [13, 41, 124]. Cholinesterase inhibitors (ChEI) act by inhibiting the enzymes (acetyl- and butyryl-cholinesterase) responsible for the breakdown of acetylcholine hence increasing its availability at the synaptic cleft. In a within-subject pilot study including 13 individuals with Alzheimer’s disease and 18 healthy controls, physostigmine improved memory by optimizing extrastriate selectivity as detected by functional magnetic resonance imaging [6]. Three ChEI’s are widely available and will be discussed in this paper: donepezil, rivastigmine, and galantamine. They are considered symptomatic treatments as they improve symptoms without modifying the course of the disease.

#### Donepezil

Donepezil is a piperidine derivative that reversibly inhibits acetylcholinesterase [113]. It is very well absorbed after oral administration and reaches peak plasmatic concentration (Cmax) in 3-4 hours. Elimination half-life of donepezil is approximately 70 hours allowing once daily administration. It binds to plasma proteins in a proportion of 96%, and is metabolised by isoenzyme 2D6 and 3A4 of cytochrome P450. Starting and minimal effective dose is 5 mg once daily. Maximal recommended dose is 10 mg daily. 

A recently published Cochrane review evaluating donepezil in AD included 24 trials and 5796 participants in mild to severe stages of the disease [9]. It showed statistically significant improvement versus placebo at 24 weeks (ADAS-Cog and SIB) and 52 weeks (MMSE). There was statistically significant improvement versus placebo on the CIBIC-plus at 24 weeks. There were also statistically significant benefits on the DAD at 24 weeks, the ADCS-ADL (version for moderate to severe AD) at 24 weeks, and PDS at 52 weeks. Overall, both the doses of 5 mg and 10 mg were beneficial, with the higher dose being marginally more effective. More side-effects were reported with donepezil than with placebo. Most common side-effects were nausea, vomiting, diarrhoea, muscle cramps, dizziness, fatigue, and anorexia, and they were dose-dependent. The authors conclude: “People with mild, moderate or severe dementia due to AD treated for periods of 12, 24, 52 weeks with donepezil experienced benefits in cognitive function, activities of daily living…Study clinicians rated global clinical state more positively in treated patients, and measured less decline in measures of global disease severity.” A positron emission tomography (PET) evaluated acetylcholinesterase (AChE) activity in 14 individuals with AD before and after treatment with donepezil [14]. The results show a modest inhibition of AChE with treatment that is most apparent in the cingulated cortex, and correlates with performance on tests of executive function and attention. A single photon emission computerized tomography (SPECT) study in 51 patients with AD before and after a 10-14 month treatment period with donepezil showed differential perfusion patterns in stabilized and non-stabilized individuals (based on their MMSE scores) [115]. In non-stabilized patients, there was lower cerebral blood flow on SPECT in the lateral and medial frontal lobes, limbic lobe, lower lateral temporal lobe, and cingulated gyrus compared to the stabilized patients. The authors conclude that SPECT may be useful in evaluating the brain function response to ChEI therapy. 

#### Rivastigmine

Rivastigmine is a carbamate derivative that reversibly inhibits both acetyl- (AChE) and butyryl- (BuChE) cholinesterase [95]. It is the only ChEI with significant inhibition of BuChE. Butyrylcholinesterase is widely distributed in the central nervous system and may play a role in cholinergic function and neurodegeneration [29]. It is unclear how specific BuChE inhibition relates to rivastigmine’s clinical effect. Rivastigmine is well absorbed after oral administration and reaches Cmax in one hour. Its elimination half-life is approximately 1 to 2 hours. It binds to proteins in a proportion of 40%, is hydrolysed by esterases (including cholinesterases), and is excreted in the urine. Cytochrome P450 isoenzymes are not involved in the metabolism of rivastigmine hence minimizing drug-drug interactions. Starting dose of rivastigmine is 1.5 mg twice a day and can be gradually titrated to the maximal dose of 6 mg twice a day. The minimal effective dose is 3mg twice a day. A transdermal form of rivastigmine has been developed and is available on most markets since 2008 [10, 130]. The main objective of transdermal rivastigmine is to allow titration to the highest (and most therapeutic) doses of the medication while minimizing side effects. This is achieved by slow release of the medication into the circulation as demonstrated by a Cmax of 8 hours by transdermal route. Starting dose of transdermal rivastigmine is 5 cm^2^, and the effective and maximal dose is 10 cm^2^.

A Cochrane review evaluating rivastigmine in AD has recently been updated and includes 9 trials and 4775 patients [8]. It shows that rivastigmine at high doses (6 to 12 mg daily) is associated with statistically significant improvement versus placebo at 26 weeks on cognitive (ADAS-Cog), global (CIBIC-plus) and functional outcomes (PDS). The results are more subtle on lower doses (4 mg daily or lower), reaching statistical significance for cognitive outcomes only. More side-effects were reported with rivastigmine than with placebo and they were dose-dependent. Most common side-effects were nausea, vomiting, diarrhoea, anorexia, headache, syncope, abdominal pain and dizziness. The recently published *Investigation of transDermal Exelon in Alzheimer’s disase (IDEAL)* study compared two doses of transdermal rivastigmine with the oral form [130]. The higher 20 cm^2^ dose (not commercially available) was not associated with more clinical benefit and led to more side-effects. The smaller 10 cm^2^ dose which is the highest commercially available dose was associated with similar clinical benefit than the maximal oral dose, but with significantly less gastrointestinal side-effects. Neither dose of transdermal rivastigmine led to behavioural benefits compared to placebo. The authors of the Cochrane review conclude: “ Rivastigmine appears to be beneficial for people with mild to moderate Alzheimer’s disease…improvements were seen in the rate of decline of cognitive function, activities of daily living, and severity of dementia…”. A plasma and Cerebrospinal fluid (CSF) biomarker study evaluated the long-term ChEI inhibitory effect of rivastigmine in eleven patients with AD treated for 12 months. This uncontrolled study showed persistent inhibition of both AChE and BuChE in plasma as well as CSF [28]. This contrasts with studies with other ChEI’s which are usually associated with up-regulation of cholinesterases. This up-regulation is sometimes invoked to explain the relatively short duration of effect of these medications. However, the relative “lack” of up-regulation of ChE with rivastigmine has not been unequivocally shown to relate to more sustained clinical benefit. 

#### Galantamine

Galantamine is a tertiary alkaloid agent that reversibly inhibits AChE [103]. It also binds allosterically to nicotinic receptors enhancing cholinergic function. The clinical relevance of allosteric binding to nicotinic receptors in humans is unclear. Galantamine is rapidly absorbed after oral administration and reaches Cmax in approximately 1 hour. Elimination half-life is between 7 to 8 hours. It binds to plasma proteins in a proportion of 18% and is metabolized by isoenzyme 2D6 and 3A4 of cytochrome P450. Galantamine is commercialized as an extended-release formulation allowing once-daily dosing. Starting dose of galantamine ER is 8 mg once daily. Minimal effective dose is 16 mg daily, and maximal dose is 24 mg daily. 

A recently published Cochrane review evaluating galantamine in mild to moderate AD and Mild Cognitive Impairment (prodrome of dementia) included 10 trials and a total of 6805 participants [80]. It showed statistically significant improvement versus placebo at 24 weeks on the ADAS-Cog, and the CIBIC-plus. Functional measures (ADCS-ADL and DAD) were seldom included in these clinical trials, but they showed statistical significance in individual trials. In contrast to other clinical trials with ChEI, there was no consistent dose-response effect in doses above 8mg daily. Galantamine’s side effects are comparable to other ChEI’s and consist mainly of cholinergic gastrointestinal symptoms. The authors conclude: “…this review shows consistent positive effects for galantamine for trials of three to six months’ duration. Although there was not a statistically significant dose-response effect, doses above 8mg/d were, for the most part, consistently statistically significant.” A randomized trial of galantamine in individuals with vascular dementia and AD with a cerebrovascular contribution (so-called “mixed dementia”) showed statistically significant benefits versus placebo on the ADAS-Cog, the CIBIC-plus, and the DAD [40]. The results of this trial has led a group of experts to recommend galantamine as “a treatment option for mixed AD with cerebrovascular disease” (Grade B, Level I recommendation) [12]. A PET study evaluated the effects of galantamine treatment on AChE and nicotinic receptor binding in 18 patients with mild AD over 12 months [68]. It shows sustained cortical inhibition of AChE. Overall, there was no statistically significant change in nicotine binding. However, both cholinesterase inhibition and nicotine binding correlated positively with performance on a test of attention in these patients. A recently published study used functional magnetic resonance imaging in two tasks of visual perception in 8 individuals with AD before and after a three-month trial of galantamine [15]. It showed a decrease in the activation of the dorsal visual pathway with treatment suggestive of a more efficient processing of visual stimuli or a modification in the compensatory changes observed in AD. 

#### Comparative Studies of Cholinesterase Inhibitors

Four clinical trials compared the ChEI’s in AD and led to conflicting results [58]. A careful review of the methodology of three of these trials using the *Consolidated Standards of Reporting Trial (CONSORT) *checklist concluded that 27% to 55% of these criteria per study were inadequately reported [64]. Considering these serious methodological limitations in comparison studies, one cannot favour one ChEI over the other at this point. Nevertheless, differences in tolerability were noted in one meta-analysis and led authors to conclude that “ across studies, the frequency in which these (adverse) events were reported was generally lowest for donepezil and highest for rivastigmine” [58]. On this issue, the CCCDTD concludes: “Although all three ChEI’s available in Canada have efficacy for mild to moderate AD, equivalency has not been established in direct comparison. Selection of which agent to be used will be based on adverse effect profile, ease of use, familiarity, and beliefs about the importance of the differences between the agents in their pharmacokinetics and other mechanisms of action.” [63]. 

### Memantine

Studies have shown that enhancement of the excitatory effects of the neurotransmitter glutamate may play a role in the pathogenesis of AD (theory of “excitotoxicity”) [120]. Memantine is a N-methyl-D-aspartate (NMDA) non-competitive glutatmate receptor antagonist [70]. It is well absorbed after oral administration and reaches Cmax in 3 to 8 hours. Elimination half-life is 60-80 hours. It binds to proteins in a proportion of 45%, and is almost completely excreted unchanged in the urine. Starting dose is 5 mg daily (in one or two doses). Minimal therapeutic dose is 10 mg daily, and maximal dose is 20 mg daily.

The Cochrane review evaluating memantine included 3 clinical trials in moderate to severe AD and three unpublished studies in mild to moderate AD [90]. In moderate to severe AD, pooled data show statistically significant benefits versus placebo at 6 months on measures of cognition (SIB), function (ADCS-ADL severe version), and global impression (CIBIC-plus). In mild to moderate AD, analyses show statistically significant benefits on measures of cognition (ADAS-cog) that do not translate into significant differences on measures of global impression and function. Memantine is generally well tolerated. Some studies suggest a reduced frequency of agitation and agressivity versus placebo, although the meta-analysis suggests an overall increased risk of agitation compared to placebo. The authors conclude: “Memantine has a small beneficial effect at six months in moderate to severe AD.” In most countries where it is available, memantine is approved for treatment of moderate to severe AD. A clinical trial compared the added efficacy of memantine in patients with moderate to severe AD receiving stable doses of donepezil [122]. It shows additional benefits of memantine versus placebo on the SIB, ADCS-ADL, and CIBIC-plus. Theses results suggest that memantine has an additive effect on ChEI’s in moderate to severe AD. A randomized placebo-controlled study is under way in Canada to determine if memantine reduces the incidence and severity of agitation and agressivity.

### Cost-Effectiveness of Symptomatic Treatments

Relatively few clinical trials have evaluated cost-effectiveness of pharmacological treatment in AD. The Cochrane review on donepezil considered the results of the two trials allowing cost-benefit evaluation and concluded: “There is some evidence that use of donepezil is neither more nor less expensive compared with placebo when assessing total health care resource cost.”[9, 46]. Several cost-effectiveness modeling studies have shown results in favour of ChEI [45]. These analyses are based mainly on the cognitive, functional and behavioural benefits provided by these medications that eventually allow delaying long-term institutionalisation. A significant portion of excess cost associated with AD is due to hospitalisations and post-acute care and this is difficult to include in cost-effectiveness models. The AD 2000 study aimed at evaluation pharmacoeconomic benefits of ChEI’s in AD. The main objectives of this non-industry sponsored clinical trial were to assess the effects of donepezil treatment on delaying institutionalisation and functional deterioration in mild to moderate AD [26]. It showed statistically significant benefits on cognitive and functional measures that did not lead to a delay in institutionalisation and functional deterioration. The authors of this study concluded: “donepezil is not cost-effective, with benefits below minimally relevant thresholds.” Unfortunately, this study suffers from several serious methodological limitations including a small number of individuals at the start of the trial compromising the power of analyses, repeated washout periods during follow-up, and significant attrition of patients with follow-up. Secondary analyses of other clinical trials and observational data have also shown results favouring pharmacological treatment. For example, secondary analyses of clinical trials with donepezil and galantamine show that the magnitude of “exposure” to the drug in terms of doses and duration is associated with a delay in institutionalisation [43, 53]. Two observational studies of patients evaluated and treated in AD research centers show that treatment with ChEI’s delays institutionalisation [78,79]. Memantine provides an additional benefit to ChEI in one study [78]. These results are encouraging but need to be validated in well-designed randomised clinical trials prospectively considering all aspects of resource utilization and indirect costs leading to a reliable and satisfying cost-effectiveness analysis. 

### Summary Of Clinical Benefit From Symptomatic Treatments and Recommendations

Overall, the benefits of symptomatic treatments in AD are modest. Some authors argue that the magnitude of this benefit, though statistically significant, is marginal at best and may even be difficult to detect, measure and quantify clinically [69, 100]. A metanalysis of 20 studies with ChEI’s specifically evaluated measurability and quantification of clinical benefit [104]. This study considered two measures of Effect Size (ES) as indicators of the magnitude of response leading it to be clinically detectable. Effect Size is determined by calculating the absolute difference in effect between the treatment and placebo groups, in relation to the variance of the given measure. Effect size is usually considered mild if between 0.2 and 0.4 and moderate when it falls between 0.41 and 0.7. The analyses show that median ES for ADAS-Cog varies from 0.15 to 0.47 depending on doses of ChEI. The same values were obtained for ES on the CIBIC-plus. The author concludes that ChEI’s produce benefits that are mild to moderate, which are dose-dependent, and more importantly, reproducible across clinical trials. Another metanalysis of 16 studies evaluated *Numbers Needed to Treat (NNT)* for clinical benefits and *Numbers Needed to Harm (NNH)* for side-effects of ChEI’s [73]. This study shows that 12 patients would need to be treated to observe minimal improvement or better on measures of cognition and global evaluation. The NNT for stabilisation or improvement is 7. NNH for one additional patient to experience an adverse event is 12.These figures compare very favourably to other well accepted interventions in medicine [47]. How do these results translate into clinical practice? In mild to moderate AD, clinical trials usually produce maximal improvement on cognitive measures after three months of treatment, but allow stabilisation of cognition for a period varying between 9 and 12 months. On measures of global impression, maximal improvement is observed at 3 months, and stabilisation usually lasts for about 6 months. On measures of function, the response usually observed is stabilisation rather than improvement. Functional capacities lost (for example managing finances or driving) are seldom recuperated with symptomatic treatment. 

Published guidelines on symptomatic treatment for AD vary in the strength of their recommendations. Certain consensus guidelines consider at least a trial of ChEI and/or memantine as a standard approach in the pharmacological treatment of AD. These include the recommendations from the *American Academy of Neurology* [39] , the *British Association of Psychopharmacology* [20], the *American Association for Geriatric Psychiatry* [82], *Recommendations for Best Practices in the Treatment of AD in Managed Care* [47], and the *Canadian Consensus Conference of Diagnosis and Treatment dementia* [63]. For example, the *CCCDTD* states that “all three cholinesterase inhibitors available in Canada are modestly efficacious for mild to moderate AD. They are all viable options for most patients with mild to moderate AD” (Grade A, Level I recommendations). The *AAN* recommends that: “cholinesterase inhibitors should be considered in patients with mild to moderate AD (Standard), although studies suggest a small average degree of benefit.” The consensus statement from the *British Association for Psychopharmacology* states: “There is type 1a evidence for the efficacy of memantine in the treatment of moderate to severe Alzheimer’s disease”. Conversely, the *National Institute for Health and Clinical Excellence (NICE)* in the UK made the following recommendations based on a cost-effectiveness evaluation of ChEI’s in AD: “The three acethylcholinesterase inhibitors donepezil, galantamine, and rivastigmine are recommended as options in the management of patients with Alzheimer’s disease of moderate severity only.” [93]. They add: “Memantine is not recommended as a treatment option for patients with moderately severe to severe Alzheimer’s disease except as part of well designed clinical studies”. The clinical practice guidelines from the *American College of Physicians and the American Academy of Family Physicians* conclude : “Clinicians should base the decision to initiate a trial of therapy with a cholinesterase inhibitor or memantine on individualized assessment.” [99, 100]. This assessment should take into consideration the “clinically marginal” benefits, the heterogeneous and unpredictable response in an individual patient, the potential for side-effects, and stage of the disease. 

### Drug Utilisation Studies of Pharmacological Treatments in AD

Two observational administrative database studies evaluated persistence with the three available ChEI in two Canadian provinces [61, 85]. The study from Quebec included 18748 individuals and the study from Ontario 5622 individuals, all aged 65 and older. Persistence rates at one year varied between 40% and 54% among individual ChEI’s in the Ontario study. In the Quebec study, the overall persistence rate for the drug class was 40% at one year. Both studies showed better persistence with galantamine as compared to donepezil and rivastigmine. The reason for this finding is unclear, but the reproducibility of this result across two different provinces is intriguing. Two studies from the US evaluated persistence with donepezil and rivastigmine [117, 121]. While mean duration of use were well below one year for both drugs, there was no difference in persistence between the two. Among the hypothesis evoked to explain these disappointingly low rates of persistence with ChEI’s are: tolerance, lack of familiarity with the drugs by primary care physicians, and unrealistic expectations by physicians, patients and their caregivers. Tolerance to these medications can be optimized by starting at low doses and slowly and individually titrating in no less than four-week interval periods. Alternate routes of administration such as the transdermal formulation of rivastigmine can significantly enhance tolerance by minimizing usual cholinergic side-effects. Alzheimer’s disease is a progressive neurodegenerative disease and available treatments do not modify progression of the process. Expectations with ChEI treatment need to be adapted to these realities. In that sense, even stabilisation of clinical symptoms for 6 to 12 months is considered an acceptable response to treatment. Slower deterioration for individual patients is more challenging to establish in clinical practice than in clinical trials where data from placebo groups are readily available for comparison. Clinically, an individual patient’s deterioration can be compared to published data on the natural history of progression of untreated AD [47]. Published recommendations are vague as to the determinants of the duration of pharmacological treatment in AD and the specific criteria for discontinuation. 

## FUTURE TREATMENTS

### Dimebon (Latrepirdine)

Latrepirdine is a non-selective anti-histaminic agent that has been used as an anti-allergic medication in Eastern Europe. *In vitro* and in animal models of AD, it has been shown to have mitochondrial stabilizing properties leading to improvement in neuronal function and inhibition of cell death [38]. In a single-country multi-site randomised clinical trial, latrepirdine was shown to be significantly superior to placebo on measures of cognition (ADAS-cog and MMSE), global evaluation (CIBIC), function (ADCS-ADL) and behaviour (NPI) at 26 weeks. It was relatively well tolerated, the most common adverse events being dry mouth and depressed mood. Several large scale phase III randomised clinical trials with latrepirdine are ongoing. If these trials replicate the findings of the initial study, latrepirdine may very well be the next pharmacological agent to be commercialized for symptomatic treatment of AD. 

### Other Treatments

All currently available treatment options for AD are symptomatic and do not modify natural progression of the disease. Disease-modifying approaches are constantly being explored. Several agents under study target the amyloïd cascade. They can be divided into three general categories: agents aiming at decreasing the production of Aβ, at reducing its aggregation, or at increasing its clearance [57, 71]. Agents that can decrease production of Aβ include gamma-secretase inhibitors [48, 116] or modulators [129] as well as alpha-secretase activators [76]. For Aβ to aggregate into insoluble toxic plaques, it needs to bind to metallic ions such as copper and zinc. Chelators inhibiting this binding such as clioquinol [101] have been proposed and studied in AD. Cyclohexanehexol [88] inhibits aggregation of Aβ into high molecular weight toxic oligomers and has been shown to reverse pathological and clinical hallmark of AD in animal models. Recently completed, but yet unpublished, phase III clinical trials with the gamma-secretase modulator flurizan and the amyloid-binding inhibitor tramiprosate showed largely negative results on primary end-points. Optimizing clearance of Aβ and its oligomers by immunization seemed very promising in animal models of AD [87]. A pilot study in patients with AD lead to an unexpected high rate of meningoencephalitis leading to interruption of the trial [96]. Neuropathologic findings in some patients enrolled in this study show a clear association between high pre-mortem serum antibody response and clearance of amyloid deposits in the brain cortex. However, there was no association between antibody response, amyloid deposition, and clinical findings [11, 65]. The immunologic approach in AD is still being tested in trials of passive immunization with intravenous immunoglobulins and monoclonal antibodies against Aβ [132]. Several of the agents described are currently in phase II-III clinical trials.

Inhibition of protein tau phosphorylation is also being explored in animal models and pilot human studies in AD. Agents in this category include glycogen synthase kinase 3-β (GSK3-β) inhibitors such as lithium [94] and valproate [134], and microtubule stabilizing agents such as paclitaxel [133]. A recently completed but unpublished phase III clinical trial with valproate is negative. 

Other agents being tested target oxidative stress in AD, neuroinflammation, cholesterol metabolism and the neuroendocrine pathways [22, 71]. 

Development of disease-modifying treatments in AD faces many challenges that will need to be considered in future research and clinical trials. Novel pharmacological agents will need to cross the blood-brain barrier and be readily bioavailable to the central nervous system. Several of the secretases being targeted have numerous substrates some of which are required for normal function. Hence, total inhibition rather than modulation may lead to unacceptable adverse events. Transferring results of animal model research to humans has been fraught with obstacles as demonstrated by the immunization trials. Finally, methodology in clinical trials will need to be adapted to detect disease-modifying effect, and this may lead to longer and more expensive studies. 

## CONCLUSIONS

In conclusion, AD is a common and costly disease. Currently several symptomatic treatments are available that provide mild benefits that are nevertheless dose-dependent, clinically detectable, and reproducible across clinical trials. Sustained use of these medications needs to be improved for optimal benefit. This can be achieved by tailoring doses and titration to the individual patients to enhance tolerance, and mostly, setting realistic expectations in face of a neurodegenerative disease and symptomatic treatments. Several agents under study are potentially disease-modifying and may significantly alter the otherwise inexorable deterioration in AD. It is such treatments that will significantly improve clinical symptoms of the disease and lead to robust pharmacoeconomic benefits such as the reduction of resource utilisation. However, when expensive disease-modifying agents will become available, they will present the clinician with many clinical and ethical challenges as to their indications, decisions to combine or discontinue treatments. These challenges will need to be considered in future clinical trials. 

## Figures and Tables

**Table 1:** Pharmacologic Properties of Widely Approved Alzheimer’s Disease Treatments

Name	Mechanism of Action	Elimination Half-life	Protein Binding	Metabolism	Starting Dose	Effective Dose	Maximal Dose
***Donepezil***	.Acetylcholinesterase inhibitor .Piperidine derivative	70 hours	96%	.Hepatic .Cytochrome P450 – 2D6 and 3A4	5mg once daily	5mg once daily	10 mg once daily
***Rivastigmine (Oral)***	.Acetylcholinesterase inhibitor .Butyrylcholinesterase inhibitor .Carbamate derivative	1-2 hours	40%	Urinary	1.5 mg twice daily	3.0 mg twice daily	6 mg twice daily
***Rivastigmine (Transdermal)***	.Acetylcholinesterase inhibitor .Butyrylcholinesterase inhibitor .Carbamate derivative	1-2 hours	40%	Urinary	5 cm^2^	10 cm^2^	10 cm^2^
***Galantamine ER***	.Acetylcholinesterase inhibitor .Nicotinic allosteric agonist .Tertiary alkaloid	7-8 hours	18%	.Hepatic .Cytochrome P450 – 2D6 and 3A4	8 mg once daily	16 mg once daily	24 mg once daily
***Memantine***	.N-Methyl-D-Aspartate (NMDA) non-competitive receptor antagonist	60-80 hours	45%	Urinary	5 mg daily (in 1 or 2 doses)	10 mg daily (in 1 or 2 doses)	20 mg daily (in 1 or 2 doses)
